# Brain Aromatase and the Regulation of Sexual Activity in Male Mice

**DOI:** 10.1210/endocr/bqaa137

**Published:** 2020-09-10

**Authors:** David C Brooks, John S Coon V, Cihangir M Ercan, Xia Xu, Hongxin Dong, Jon E Levine, Serdar E Bulun, Hong Zhao

**Affiliations:** 1 Division of Reproductive Science in Medicine, Department of Obstetrics and Gynecology, Feinberg School of Medicine, Northwestern University, Chicago, Illinois; 2 Protein Characterization Laboratory, Cancer Research Technology Program, Frederick National Laboratory for Cancer Research, Leidos Biomedical Research Inc., Frederick, Maryland; 3 Department of Psychiatry & Behavioral Sciences, Feinberg School of Medicine, Northwestern University, Chicago, Illinois; 4 Wisconsin National Primate Research Center, Department of Neuroscience, School of Medicine and Public Health, University of Wisconsin, Madison, Wisconsin

**Keywords:** aromatase, estrogen, testosterone, brain, sexual behavior

## Abstract

The biologically active estrogen estradiol has important roles in adult brain physiology and sexual behavior. A single gene, *Cyp19a1*, encodes aromatase, the enzyme that catalyzes the conversion of testosterone to estradiol in the testis and brain of male mice. Estradiol formation was shown to regulate sexual activity in various species, but the relative contributions to sexual behavior of estrogen that arises in the brain versus from the gonads remained unclear. To determine the role of brain aromatase in regulating male sexual activity, we generated a brain-specific aromatase knockout (bArKO) mouse. A newly generated whole-body total aromatase knockout mouse of the same genetic background served as a positive control. Here we demonstrate that local aromatase expression and estrogen production in the brain is partially required for male sexual behavior and sex hormone homeostasis. Male bArKO mice exhibited decreased sexual activity in the presence of strikingly elevated circulating testosterone. In castrated adult bArKO mice, administration of testosterone only partially restored sexual behavior; full sexual behavior, however, was achieved only when both estradiol and testosterone were administered together. Thus, aromatase in the brain is, in part, necessary for testosterone-dependent male sexual activity. We also found that brain aromatase is required for negative feedback regulation of circulating testosterone of testicular origin. Our findings suggest testosterone activates male sexual behavior in part via conversion to estradiol in the brain. These studies provide foundational evidence that sexual behavior may be modified through inhibition or enhancement of brain aromatase enzyme activity and/or utilization of selective estrogen receptor modulators.

Estrogens are considered to be critical for the regulation of reproductive function in both sexes (1). Estrogens have important organizational and functional roles in brain development as well as in adult physiology and behavior (2-5). During development, estrogen is involved in axon and dendrite growth and neuronal differentiation (2). In adults, estrogens modulate neurotransmitter production and release, enzyme activity, membrane potential, dendritic arborization, and synaptogenesis (6, 7). Estrogens exert their biological function primarily through 2 nuclear estrogen receptors: ERα and ERβ. Interestingly, both receptors are expressed in neurons and glia (8).

Aromatase, the enzyme that catalyzes the conversion of androgens to estrogens, is encoded by a single gene, *Cyp19a1* (9, 10). In male mice, aromatase is expressed mainly in the testis and brain (11); however, the distinct roles of brain versus testicular aromatase in brain function are currently unknown. In the vertebrate brain, aromatase is mainly expressed in neurons of the hypothalamus and amygdala (12). Neuronal aromatase expression is tightly controlled via a highly conserved brain-specific promoter I.f (13-15). In the hypothalamus, aromatase is expressed primarily in the preoptic and the ventromedial nuclei (12, 16), regions that have been implicated in the regulation of reproductive functions and behavior of both sexes (13-15, 17, 18). Aromatase-expressing neurons within the male posterodorsal medial amygdala regulate male aggression, but not other sexually dimorphic behaviors, such as marking, singing, or mating (19). Most recently, aromatase-expressing neurons within the principal component of the bed nucleus of stria terminalis, a limbic center and the extended amygdala, have been found to be required for sexual preference, mating, and aggression (20). Thus, local estrogen production via aromatase expression in these regions of the brain may be critical for normal reproductive function and behavior.

Aromatase deficiency, while very rare, has been reported in humans. The few aromatase-deficient men studied were reported to be heterosexual, with chronically elevated luteinizing hormone (LH), follicle-stimulating hormone (FSH), and testosterone (T) (21-24). Studies evaluating the effects of estrogen therapy on libido in aromatase-deficient men reported significant improvements in the frequency of sexual activity following treatment, suggesting that estrogens act either alone or synergistically with androgens to regulate sexual behavior (21, 25). Compared with wild-type (WT) mice, male whole-body aromatase knockout (ArKO) mice exhibit overall decreased sexual activity, which becomes more prominent with advancing age (26-30). These mice also have increased circulating LH, FSH, and T, similar to aromatase-deficient men (24, 31). As in humans, administration of exogenous estradiol (E_2_) partially restored sexual activity in whole-body ArKO mice (26). Moreover, brain-specific overexpression of the human aromatase gene can nearly restore sexual activity in ArKO mice (32). These observations are suggestive that brain aromatase is involved in regulating T-dependent gonadotropin regulation and sexual behavior in males. However, it has remained unclear whether E_2_ secreted as a result of testicular aromatase or the conversion of circulating T to E_2_ by brain aromatase is responsible for regulating sexual activity in male mice.

Three previously generated whole-body ArKO models show phenotypic changes suggesting vital roles of E_2_ in male reproductive function (27, 30, 31). These complete knockout models, however, block E_2_ production in both gonadal and extragonadal tissues and its secretion into the circulation (31). Consequently, these models do not allow for investigation of the tissue-specific effects of aromatase. We created a novel brain-specific conditional aromatase knockout mouse (bArKO) and compared sexual activity and hormone levels to those of a newly generated whole-body total aromatase knockout mouse (tArKO) and WT and heterozygous littermate controls. We ascertained whether brain aromatase is essential for normal sexual behavior in male mice and whether the alterations in sexual behavior are caused by estrogen deficiency.

## Materials and Methods

### Generation of Arom^fl/fl^ mice

**Figure 1.**
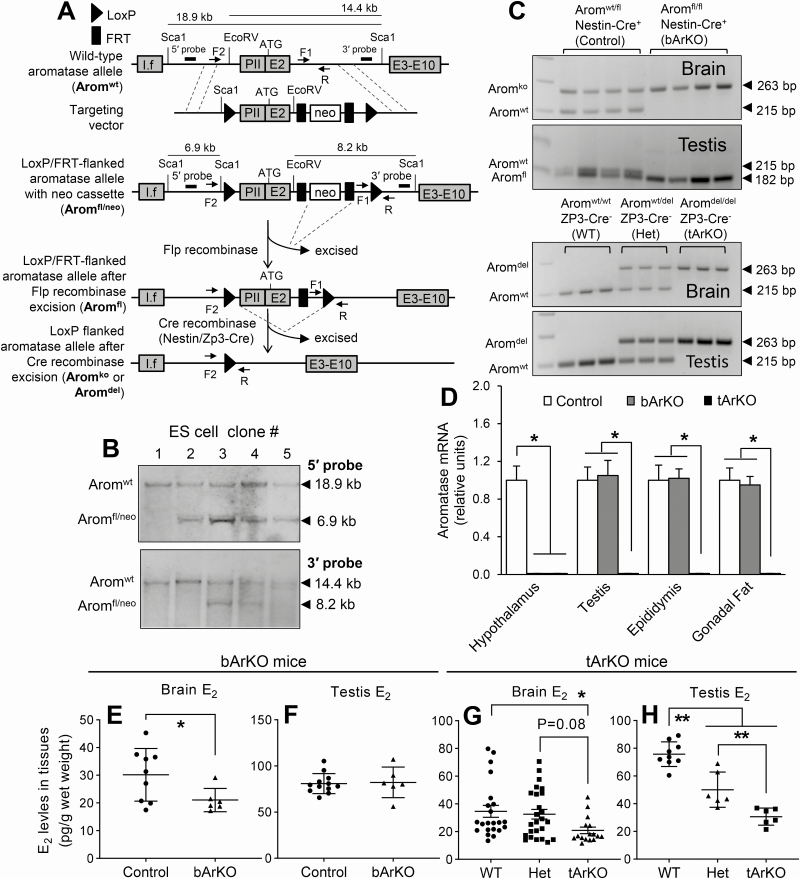
**Generation and characterization of bArKO and tArKO mice.** (A) Schematic demonstrating generation of the floxed murine aromatase gene and its recombination after expression of recombinases. The upper diagram shows the targeting construct used to introduce loxP and FRT sites into the WT aromatase gene; restriction enzyme sites (Sca1 and EcoRV), the location of 5′ and 3′ probes, and the position of the primers (F1, F2, and R) for the PCR analysis are indicated. The middle diagram shows the targeting construct after homologous recombination. The aromatase allele together with the FRT-flanked neo cassette was flanked by LoxP sites (Arom^fl/neo^). The lower diagram indicates the floxed aromatase alleles after transient Flp recombinase expression with subsequent deletion of the neo cassette (Arom^fl^). Nestin-Cre recombinase expression with knockout of the flanked region of the aromatase gene (Arom^ko^) in the brain generated bArKO mice, and Zp3-Cre recombinase expression with complete deletion of the flanked region of the aromatase gene (Arom^del^) in whole body generated tArKO mice. I.f, a brain-specific exon 1. PII, a gonad-specific first exon. E2, exon 2. E3-E10, exon 3 to exon 10. (B) Southern blot analysis of different ES cell clones with the 5′ probe and 3′ probe after transfection with the LoxP/FRT flanked targeting construct. Clones #3 and #4 showed the expected bands of 18.9 kb (Arom^wt^ allele) and 6.9 kb (Arom^fl/neo^ allele) using the 5′ probe and the expected bands of 14.4 kb (Arom^wt^ allele) and 8.2 kb (Arom^fl/neo^ allele) using the 3′ probe, indicating that 1 copy of the WT aromatase gene was replaced by the targeting construct. (C) PCR analysis of DNA prepared from brain and testis of bArKO and tArKO mice. bArKO mice produced a 263-bp band (Arom^ko^ allele) in brain indicating a recombination event in brain and a 182-bp band (Arom^fl^ allele) in testis indicating a loxP-flanked aromatase gene without recombination in testis. Aromatase heterozygous control mice contained an Arom^wt^ allele (a 215-bp band) and an Arom^ko^ allele in brain, and an Arom^wt^ allele and an Arom^fl^ allele in testis. tArKO mice contained only the Arom^del^ allele (a 263-bp band) in brain and testis suggesting that recombination occurred in brain and testis. Aromatase heterozygous control mice (Het) contained an Arom^wt^ allele and an Arom^del^ allele in brain and testis. WT mice only contained the Arom^wt^ allele. (D) Real-time quantitative PCR demonstrating mRNA levels of aromatase-expressing tissues in control, bArKO, and tArKO mice. GAPDH mRNA levels served as loading controls. n = 5. E_2_ levels in the brain (E and G) and testis (F and H) of bArKO and tArKO mice were measured by the LC-MS/MS assay. 2-tailed Student *t* test or 1-way ANOVA with Tukey multiple comparison test, *P < 0.05, **P < 0.01. n = 9-12 for controls and n = 6 for bArKO mice; n = 21 for WT, n = 25 for Het, and n = 16 for tArKO mouse brain; n = 9 for WT, n = 6 for Het, and n = 6 for tArKO mouse testis.

To generate the targeting vector, a 14.8-kb aromatase (*Cyp19a1)* genomic DNA fragment was subcloned from a BAC clone (RP23-236O14) containing the full length of the aromatase gene (33). A targeting vector was then constructed to delete 4.9-kb of promoter region and 0.6-kb of gonad-specific first exon (PII), exon 2, and partial intron 2 of the aromatase gene. Both the transcription start site and the translation initiation site were included in this targeting vector for deletion upon expression of Cre protein. A neomycin resistance (*neo*) gene flanked by 2 FRT sites was introduced into intron 2 of the gene. Additionally, 2 loxP sites were introduced either at 5-kb upstream of exon 2 or downstream of the FRT-neo-FRT cassette within intron 2 for targeted gene deletion (Fig. 1A). Mouse embryonic stem (ES) cells were electroporated with this vector and selected in the presence of G418; homologous recombinant clones were identified and confirmed by Southern blot analysis. 5´and 3´ probes were used to identify the presence of hybridization bands corresponding to the loxP-flanked aromatase (Arom^fl/neo^) allele or wild-type aromatase (Arom^wt^) allele (Fig. 1B). The correctly targeted ES cells (clones #3 and #4) were then transiently transfected with an Flp recombinase expression plasmid to delete the FRT-flanked neo cassette, leaving a single FRT site that was identified by polymerase chain reaction (PCR). The resulting ES clone DNA, carrying the floxed aromatase gene, was used to generate 2 chimeric mice that successfully transmitted the gene in the germ line. Mice homozygous for the floxed aromatase gene (*Arom1*^*fl/fl*^) were born at the expected Mendelian ratios and presented with no obvious abnormalities. The presence or absence of the floxed allele of aromatase was confirmed by PCR analysis [forward primer 1 (F1): 5´-TCCAAGTCAGAAAGGTTGGTG-3´, forward primer 2 (F2): 5´- ATGGAGTAGGCAGGGTCAAGT-3´, reverse primer (R): 5´- GAGAAAGGATAAACGCAAGCA-3´]. All mice were kept on a C57BL/6J background.

### bArKO and tArKO mouse generation and maintenance

Transgenic Nestin-Cre mice (#3771) and Zp3-Cre mice (#3651) on a C57BL/6J background were obtained from The Jackson Laboratory and crossed with *Arom*^*fl/fl*^ mice to generate bArKO or tArKO mice, respectively. Mice were housed under a 14-10 light-dark cycle and provided *ad libitum* water and chow. Husbandry and all animal procedures were approved by and conducted in accordance with guidelines established by the Institutional Animal Care and Use Committee at the Northwestern University.

Serum and tissue hormone levels measured by radioimmunoassay (RIA), enzyme-linked immunosorbent assay (ELISA), and liquid chromatography-tandem mass spectrometry (LC-MS/MS)

Serum was collected via retro-orbital bleeding from 8- to 26-week-old bArKO, tArKO, and littermate control mice. All tissue and serum samples were collected from mice between 10 am and noon to minimize assay variability due to daily hormone fluctuations. Serum levels of FSH (Cat#:AFP1760191; A.F. Parlow National Hormone and Peptide Program; RRID:AB_2665512) (34) and LH (Cat#:518B7; Dr. Janet Roser, Department of Animal Science, University of California, Davis, CA; RRID:AB_2665514) (35) were measured by RIA and serum levels of T (Cat#:IB79106; Immuno-Biological Laboratories, Minneapolis, MN; RRID:AB_2814981) (36) and androstenedione (A4) (Cat#:11-ANRHU-E01; Alpco Diagnostics, Salem, NH; RRID:AB_2756382) (37)) in bArKO mice were measured by ELISA at the Ligand Assay and Analysis Core of the University of Virginia Center for Research in Reproduction. Serum and brain and testis tissue levels of E_2_, brain and testis levels of T and A4, and tArKO serum levels of T and A4 were determined by the stable isotope dilution high performance LC-MS/MS at Frederick National Laboratory for Cancer Research, as described previously (38-43). In brief, Omni Bead Ruptor (Omni International, Kennesaw, GA) was used for brain and testis tissue sample homogenization. Stable-isotope–labeled steroid internal standards were added to serum samples and brain and testis tissues. After tissue sample homogenization, all samples underwent liquid-liquid extraction with dichloromethane/hexane (1:1) and derivatization with dansyl chloride. The stable-isotope dilution high performance LC-MS/MS analysis was performed using a Thermo TSQ Quantiva triple quadrupole mass spectrometer (Thermo Fisher, San Jose, CA) coupled with a NexeraXR LC system (Shimadzu Scientific Instruments, Columbia, MD). Both the chromatography and mass spectrometry were controlled by Xcalibur software (Thermo Fisher, San Jose, CA). The stable-isotope labeled A4, T, and E_2_ were used in this assay to account for potential losses during sample preparation and analysis, which included: androstenedione-2,3,4-^13^C_3_ (^13^C_3_-A4) and testosterone-2,3,4-^13^C_3_ (^13^C_3_-T) purchased from Cerilliant Corporation (Round Rock, TX); and 17β-estradiol-13,14,15,16,17,18-^13^C_6_ (^13^C_6_-E_2_) obtained from Cambridge Isotope Laboratories, Inc. (Andover, MA).

### Steroid LC-MS/MS assay validation

The calibration curves for the unconjugated A4, T, and E_2_ measured in this study were linear over 1000-fold concentration ranges with linear regression correlation coefficients greater than 0.998. The lower limit of quantitation (LLOQ) in the present assay was defined as the lowest concentration of a steroid in a sample that could be determined with acceptable precision and accuracy specified as intra- and inter-batch coefficients of variation (CV) within 15% and measured analyte values within 85% to 115% of known target values under the conditions of the described assay method (44, 45). The LLOQs for our serum steroid LC-MS/MS assay were 10 pg/mL for A4 and T and 0.4 pg/mL for E_2_. The LLOQs for the tissue steroid LC-MS/MS assay were 10 pg/g wet weight for A4 and T and 0.4 pg/g wet weight for E_2_. The steroid LC-MS/MS assay accuracy was measured as the percent recovery of the known added amount in spiked samples (44, 45). High specificity of this assay was achieved through triple-quadrupole mass spectrometry selective reaction monitoring coupled with high resolution chromatography. No samples from the current study had undetectable levels for any of the steroids measured. Laboratory CVs of blinded duplicated quality control samples within and across batches were <5% and <15% respectively for all steroids measured.

### DNA extraction and analysis by PCR and Southern blot

Tissues were thawed and placed in 500 mL DNA extraction buffer (0.1M NaCl, 20 mM TRIS-EDTA, 0.5% SDS) and manually disrupted in TissueLyser for 5 minutes. Tissue was further digested with proteinase K (1 mg/mL) for at least 12 hours at 55 °C. DNA was extracted using standard phenol/chloroform/isoamyl alcohol protocols. PCR analysis was performed using 10 ng of DNA from tissue samples. PCR products were resolved by gel electrophoresis in 3% agarose. Three sets of primers (F1, F2, and R, shown above) were designed to determine the presence of the 3 distinct aromatase alleles representing 3 PCR products: a 263-bp Arom^ko^ or Arom^del^ allele, a 215-bp Arom^wt^ allele, and a 182-bp Arom^fl^ allele. Southern blot analysis was performed as described (46). In brief, genomic DNA isolated from ES cells was digested overnight by using Sca1 or EcoRV and hybridized with ^32^P-labeled 5´ probe or 3´ probe (Amersham Rediprime DNA labeling system, GE Healthcare, Pittsburgh, PA).

### RNA analysis

Tissues to be used for RNA isolation were flash-frozen in liquid nitrogen and stored at −80 °C until further use. Total RNA was extracted from various tissues using TRI reagent (Sigma-Aldrich, St. Louis, MO) per the manufacturer’s instructions. RNA integrity was assessed following electrophoresis on a 1% formaldehyde gel. Reverse transcription (RT) was performed using 1 µg of total RNA with qScript cDNA SuperMix (Quanta Biosciences, Gaithersburg, MD). Real-time quantitative PCR was performed in triplicate employing 1 µL of cDNA using a Power SYBR Green Master Mix (Applied Biosystems, Foster City, CA) on an Applied Biosystems 7900 Sequence Detection System as previously described (11). The threshold cycle (Ct), defined as the fractional cycle number at which the fluorescence passes a fixed threshold, was used to calculate relative mRNA units as described previously (11). RNA samples were normalized to GAPDH, an endogenous control. Template-free and RT-negative controls were used to ensure reaction specificity and the absence of genomic DNA.

### Hormone replacement

Hormone pellets were made by Research Innovations of America (Sarasota, FL). Pellets of T (25 mg), E_2_ (0.25 mg), or cholesterol (vehicle) were made in a 60-day continuous-release formula. Experimental male mice were castrated at approximately 9 weeks of age and given replacement hormone thereafter. Pellets were implanted subcutaneously on the lateral side of the neck between the ear and the shoulder.

### Sexual behavior analysis

All sexual behavior analysis was performed in the Northwestern University Behavior Phenotyping Core. Male mice were housed individually beginning at 6 weeks of age. All experiments were performed on virgin males 12-14 weeks of age. Mating behavior was measured in 30-minute tests with unfamiliar, hormonally primed, sexually experienced FVB/NJ female mice aged 12-20 weeks. Stimulus females were ovariectomized and hormonally primed with subcutaneous injections of 10 µg of E_2_ benzoate dissolved in peanut oil 48 and 24 hours before testing, and 500 µg progesterone dissolved in peanut oil 6 hours before testing. Male mice were placed in clean, enrichment-free, 13.5” × 8” × 5.5” cages 6-8 hours before testing. Each male was tested from 2 to 6 hours following lights out. A stimulus female was placed in the cage of the male for 30 minutes, and behavior was video recorded from above for blinded scoring, as follows. We scored mounts and intromissions as male reproductive behavior. We evaluated the latency, duration, and frequency of these events. If a subject did not perform the behavior during the 30-minute test, a maximum latency of 1800 seconds was assigned for that behavior. We tested each male mouse twice, with 5 to 10 days separating consecutive trials. Each male mouse was randomly assigned a different primed female for the second analysis. We used stimulus female mice in no more than 6 different tests. Three independent blinded observers scored the videotapes (D.C.B., H.Z., and C.M.E.)

### Statistical analysis

All statistical tests were performed using GraphPad Prism software (Version 8.2.1, GraphPad, San Diego, CA). Results were reported as mean ± SEM, unless otherwise indicated. Statistically significant differences at *P* < 0.05 were determined using a 2-tailed Student *t* test, 1-way ANOVA, or 2-way ANOVA followed by the Tukey multiple comparison test.

## Results

### Generation and characterization of a brain-specific aromatase knockout mouse

Aromatase is expressed in mouse gonads, brain, and gonadal fat tissue (11). To knock out aromatase in a tissue-specific fashion, we first generated mice with a loxP-flanked aromatase gene (*Arom*^*fl/fl*^). LoxP sites in *Arom*^*fl/fl*^ mice flanked a 5.5-kb genomic region containing the proximal regulatory region and the adjacent coding region with the ATG translational start site. This floxed region comprises the gonad-specific promoter, the gonad-specific untranslated first exon (PII), the adjacent coding exon 2, and a part of intron 2 of the aromatase gene, which includes the transcription and translation start sites and the common splice acceptor site for the upstream brain promoter I.f (Fig. 1A). Recombinant ES cells contained the floxed aromatase allele with neo cassette (Arom^fl/neo^) and WT allele (Arom^wt^), which were confirmed by Southern blot analysis and used to generate 2 *Arom*^*fl/fl*^ mouse lines (Fig. 1A-1C).

We crossed Nestin-Cre mice with *Arom*^*fl/fl*^ mice to generate brain-specific aromatase knockout mice (bArKO). We also generated whole-body total aromatase knockout mice (tArKO) to be used as positive controls by crossing the same *Arom*^*fl/fl*^ mice with Zp3-Cre mice. To verify the generation of bArKO and tArKO mice, we harvested various tissues from 3-month-old male mice to assess the profile of DNA recombination and aromatase mRNA expression. PCR analysis of the aromatase gene was performed on DNA isolated from the brain and testis of bArKO, tArKO, and littermate control mice (Fig. 1C). We designed a PCR screen to differentiate between the 3 distinct aromatase alleles (Arom^wt^, Arom^fl^, and Arom^ko^/Arom^del^). Alleles missing the aromatase gene were Arom^ko^ in the brain of bArKO mice or Arom^del^ in the tArKO mice. Analysis of bArKO mice showed full recombination in the brain, with only 1 Arom^ko^ allele amplified, and no recombination in the testis, with only 1 Arom^fl^ allele amplified. Littermate control heterozygous mice (Arom^wt/fl^/Nestin-Cre^+^) showed both Arom^wt^ and Arom^ko^ alleles in the brain and both Arom^wt^ and Arom^fl^ alleles in the testis (Fig. 1C). tArKO mice had amplification of the Arom^del^ allele in both brain and testes, whereas littermate WT control mice had the Arom^wt^ allele and heterozygous (Het) control mice had both an Arom^del^ and Arom^wt^ allele in each tissue (Fig. 1C). Aromatase mRNA in the hypothalamus, testis, epididymis, and gonadal fat was analyzed by real-time quantitative PCR using tissue-specific primers (Fig. 1D). In bArKO mice, aromatase mRNA was reduced by more than 100-fold in the hypothalamus, whereas no change was detected in other aromatase-expressing tissues (testis, epididymis, or gonadal fat). As expected, aromatase mRNA was undetectable in all tissues collected from tArKO mice (Fig. 1D). Thus, the bArKO mice had full recombination at the DNA level and lacked aromatase mRNA expression in the brain, while maintaining appropriate aromatase expression in the gonads and gonadal fat.

To determine the reproductive function and fertility of bArKO mice, bArKO and control (heterozygous) males were mated with WT females. The average litter size between the controls and bArKO mice was similar. However, 100% of tArKO mice were infertile (more details in Supplemental Fig. 1, available in an online data repository (47)). Body weight was assessed weekly for bArKO, tArKO, and age-matched heterozygous control male mice from 3 to 48 weeks of age. Body weight was similar between control and bArKO mice but significantly increased in tArKO mice starting from 20 weeks of age compared with control mice (more details in Supplemental Fig. 2, available in an online data repository (47)).

### Deletion of brain aromatase alters brain estrogen production and testosterone secretion

To determine the role of brain aromatase in the regulation of tissue estrogen levels, we measured brain and testis E_2_ concentrations using LC-MS/MS (Fig. 1E and 1F). Compared to control mice, brain E_2_ levels were significantly lower in bArKO mice (Fig. 1E). However, testis E_2_ levels measured by LC-MS/MS were not significantly different between the control and bArKO males (Fig. 1F). T and A4 levels in the brain and testis did not differ between control and bArKO male mice (more details in Supplemental Fig. 3, available in an online data repository (47)).

Compared with WT littermates, E_2_ levels in the brain and testis of tArKO mice were significantly lower; compared with Het mice, a significant difference in E_2_ levels was observed only in testis but not in the brain (Fig. 1G and 1H). T and A4 levels in the brain and testis were significantly higher in tArKO mice compared with WT mice (more details in Supplemental Fig. 4, available in an online data repository (47)).

### Aromatase and estrogen in the brain are required for male sexual behavior

**Figure 2.**
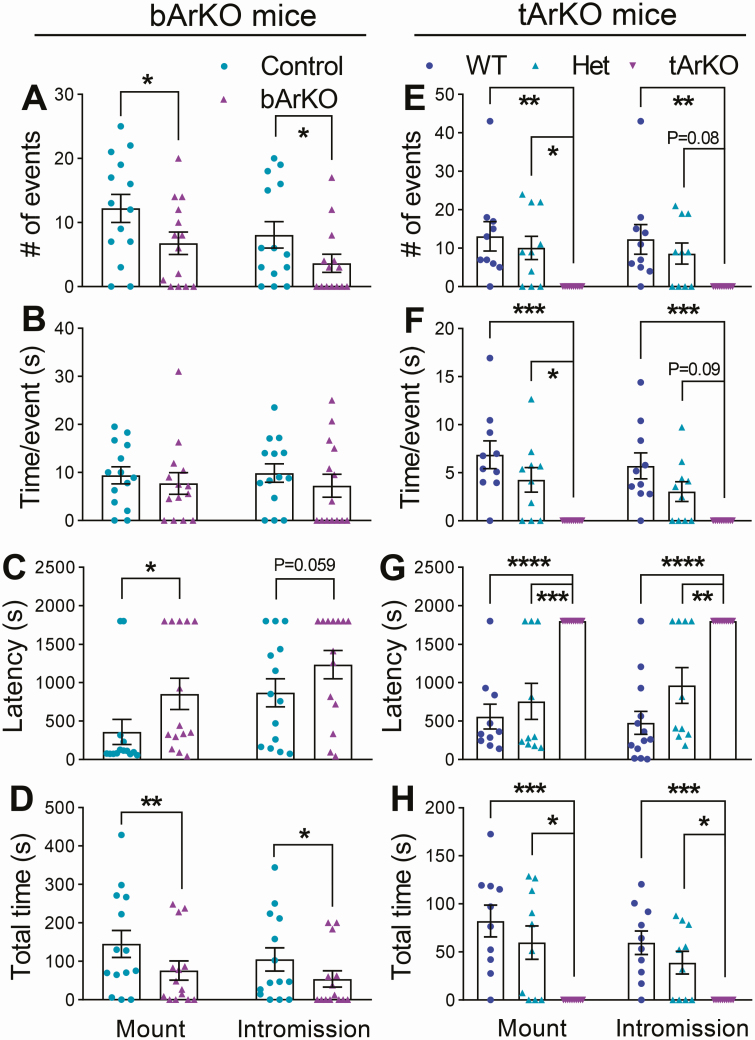
**Baseline sexual behavior of bArKO mice is decreased compared to controls.** Sexual activity was measured in 12- to 14-week-old intact (noncastrated) bArKO, tArKO, and littermate control male mice over two 30-minute trials. The interactions were monitored and videotaped for the further analysis. (A) The number of events (mount or intromission), (B) time per event, (C) first event latency, and (D) total time for mounts or intromissions in bArKO mice. n = 7-8 for bArKO mice or heterozygous littermate controls. (E) The number of events (mount or intromission), (F) time per event, (G) first event latency, and (H) total time for mounts or intromissions in tArKO mice. n = 5 for tArKO mice, Het mice, or WT littermate controls. 2-tailed Student *t* test for bArKO mice, 1-way ANOVA with Tukey multiple comparison test for tArKO mice, *P < 0.05, **P < 0.01, ***P < 0.001, ****P < 0.0001.

To determine the importance of brain aromatase and estrogens in the regulation of sexual behavior, we measured sexual activity in 12- to 14-week-old intact (noncastrated) bArKO, tArKO, and littermate control males in two 30-minute trials. The interactions were monitored and videotaped, and the videotape was scored for the time of onset, duration, and frequency of sexual behavior (mounts and intromissions). Intact bArKO mice showed a substantially and significantly lower (approximately 50% less; *P* < 0.05) total number of mounts and intromissions during these encounters compared with littermate controls (Fig. 2A). Interestingly, the average mount length or intromission length was not different in intact bArKO mice compared with controls (Fig. 2B). The average mount latency, a measure of the average time to initiate the first mount, was approximately 2-fold higher in intact bArKO mice compared with controls (*P* < 0.05; Fig. 2C). There was a trend (*P* = 0.059) toward longer intromission latency in bArKO mice versus controls. Overall, intact bArKO mice spent less total time engaged in sexual behaviors such as mounting or intromission (Fig. 2D and more details in Supplemental Movies 1 and 2, available in an online data repository (47)). Taken together, these data suggest that while there was no difference in the qualitative acts associated with sexual behavior in intact bArKO and control mice, intact bArKO mice initiated these behaviors significantly less frequently.

By comparison, the disruption of sexual behavior was more severe in intact tArKO than in intact bArKO mice, with intact tArKO mice exhibiting no sexual activity (Fig. 2E-2H and more details in Supplemental Movies 3-5, available in an online data repository (47)). Mount and intromission behaviors were not observed in 5 intact tArKO mice analyzed across a total of 10 trials (Fig. 2E). WT mice demonstrated levels of sexual activity similar to the heterozygous controls in the bArKO experiments (Fig. 2E-2H). These data suggest that aromatase is necessary for sexual activity in male mice. Testicular aromatase plays a dominant role in male sexual behavior, but brain aromatase specifically contributes to regulating the initiation and frequency of sexual activity.

### Exogenous sex steroids rescue sexual behavior in castrated bArKO male mice

**Figure 3.**
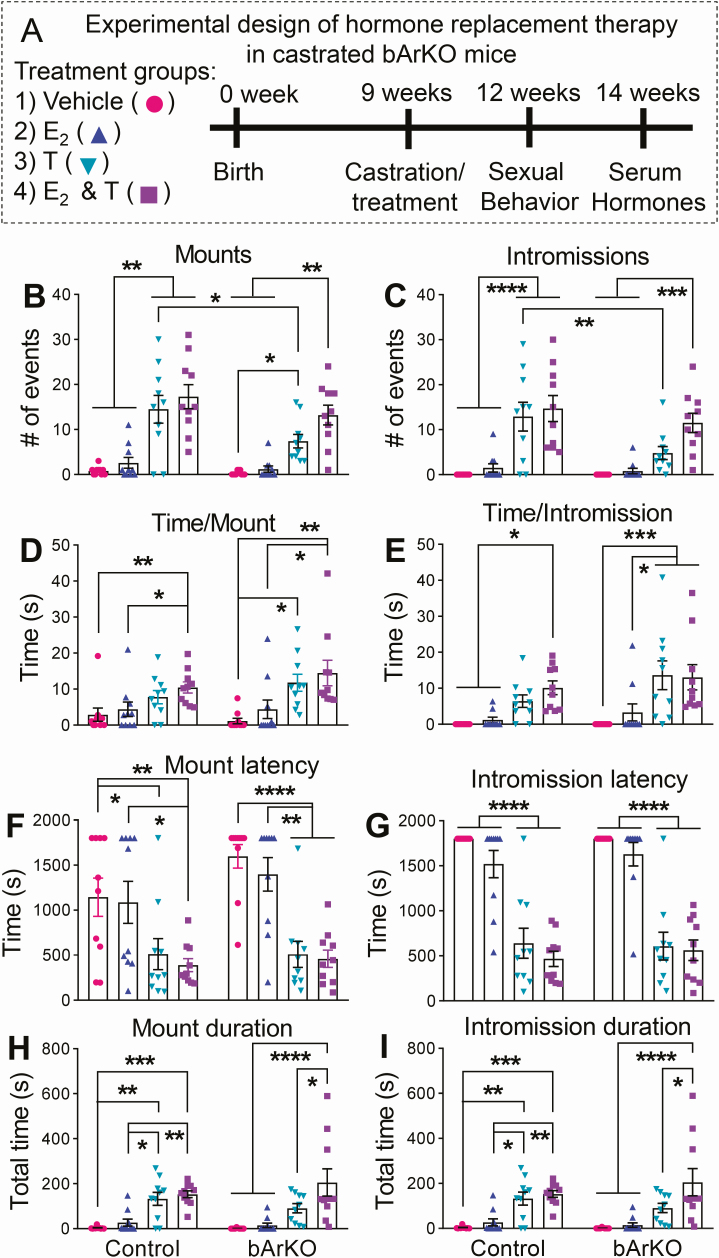
**Sexual behavior following hormone replacement in control and bArKO castrated mice.** (A) Schematic diagram depicts the schedule of hormone replacement therapy and assays. (B) The number of mounts, (C) number of intromissions, (D) time per mount, (E) time per intromission, (F) first mount latency, (G) first intromission latency, (H) total time for mounts, and (I) total time for intromissions in castrated control and bArKO mice following each hormone treatment. n = 5 mice per group. Sexual activity was assessed in each mouse twice. Two-way ANOVA with Tukey multiple comparison test, *P < 0.05, **P < 0.01, ***P < 0.001, ****P < 0.0001.

To investigate whether the lack of estrogen production in the brain caused the altered sexual behavior in bArKO mice, 20 bArKO and 20 littermate heterozygous control mice were castrated at approximately 9 weeks of age and then exogenous sex hormones were replaced via an implanted 60-day time-release pellet. Castrated mice were used for exogenously manipulating circulating hormone levels. Mice were given vehicle, E_2_ (0.25 mg), T (25 mg), or E_2_ (0.25 mg) plus T (25 mg) (n = 5/group/genotype) and the effect on sexual behavior was measured after 3 weeks of hormone replacement therapy (Fig. 3A).

Both castrated bArKO and heterozygous control mice treated with vehicle or E_2_ alone showed negligible sexual behavior (mounts/intromissions, Fig. 3B-3I). The lack of sexual behavior in castrated control mice following E_2_ treatment indicates that systemically delivered E_2_ alone is not sufficient to stimulate male sexual behavior. Control mice treated with either T or a combination of T and E_2_ showed robust sexual activity (Fig. 3B-3I). There did not appear to be a difference in the amount of sexual behavior in terms of the number of mounts or intromissions in control mice treated with either T alone or T in combination with E_2_ (Fig. 3B and 3C). Importantly, bArKO mice receiving T treatment alone showed significantly less sexual activity (number of mounts or intromissions) than control mice receiving T alone, indicating that brain E_2_ is required for full sexual activity (Fig. 3B and 3C). The average time per mount and average time per intromission (Fig. 3D and 3E) and the mount and intromission latency (Fig. 3F and 3G) were not different in bArKO mice or control mice treated with T alone or T in combination with E_2_. However, bArKO mice treated with T alone spent less time overall engaging in sexual behavior (Fig. 3H and 3I) compared with bArKO mice treated with T plus E_2_. The differences were statistically significant for both mount and intromission duration (Fig. 3H and 3I). No difference in duration of sexual behavior was seen in control mice receiving T versus T plus E_2_, possibly because T is aromatized to E_2_ in the brain in control animals receiving T only. Taken together, our findings indicate that a combination of T and E_2_ is required to rescue appropriate sexual behavior in mice with deficient E_2_ production in the brain.

### Circulating hormone levels in intact and castrated bArKO mice

**Figure 4.**
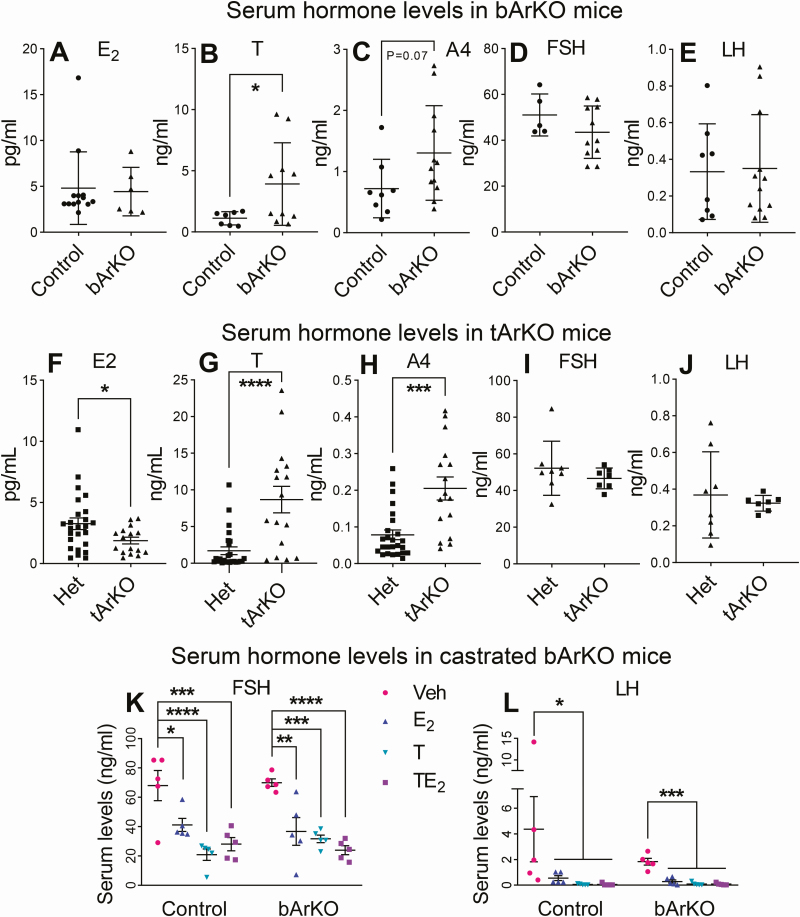
**Serum hormone levels in bArKO and tArKO mice.** Serum E_2_ (A and F), T (B and G), A4 (C and H), FSH (D and I), and LH (E and J) were measured in bArKO and tArKO mice, respectively. Serum levels from 8- to 26-week-old mice shown in panels A and F-H were measured by LC-MS/MS and serum levels from 12-week-old mice shown in panels B and C were measured by ELISA and in panels D, E, I, and J were measured by RIA. For bArKO mice, n = 13 for controls and n = 6 for bArKO mice in A, and n = 8-12 per group in B-E. For tArKO mice, n = 25 for Het controls and n = 16 for tArKO mice in F-G and n = 7-8 per group in I and J. Two-tailed Student *t* test, *P < 0.05, ***P < 0.001, ****P < 0.0001. Serum FSH (K) and LH (L) levels following steroid hormone replacement in castrated bArKO mice were measured by RIA. Castrated mice were treated with vehicle (Veh), E_2_, T, or T plus E_2_ (TE_2_) for 5 weeks. Mouse serum was collected from 14-week-old mice. One-way or 2-way ANOVA with Tukey multiple comparison test, *P < 0.05, **P < 0.01, ***P < 0.001, ****P < 0.0001, n = 5 mice per group.

Circulating E_2_ levels in bArKO mice were similar to those of controls (Fig. 4A). Note that brain E_2_ levels in bArKO mice were significantly reduced compared with controls, as expected (see Fig. 1E); on the other hand, serum T levels were significantly elevated in bArKO mice (Fig. 4B). A4 of primarily testicular origin was also elevated but did not reach statistical significance (*P* = 0.07). T and A4 elevations in bArKO may be driven by LH, which is partially suppressed by E_2_. We did not detect any differences in mean circulating LH or FSH levels in bArKO mice compared with heterozygous controls. This may be due to the inability to detect alterations in the frequency of pulsatile gonadotropins, which occur following removal of feedback inhibition (Fig. 4D and 4E). The tArKO mice had decreased circulating E_2_ levels and increased T and A4 levels, as expected (Fig. 4F-4H). We did not detect any differences in the mean circulation LH or FSH levels in tArKO mice, possibly because of their highly pulsatile release with large variances in control mice (Fig. 4I and 4J).

To further characterize the roles of exogenous sex steroid hormones in regulating sex gonadotropins, serum was collected from castrated bArKO and heterozygous control mice after completing the sexual behavior assessment and analyzed for FSH and LH (Fig. 4K and 4L). Both castrated bArKO and heterozygous controls treated with vehicle showed elevated FSH and LH (Fig. 4K and 4L). E_2_ treatment in both groups of mice led to a significant reduction in FSH and LH, indicating that E_2_ has an inhibitory effect on gonadotropin release in male mice. Interestingly, T alone also decreased FSH and LH in both control and bArKO mice. This indicates that T aromatization in the brain is not required for inhibition of gonadotropin secretion in male mice. Addition of E_2_ to T treatment did not alter the gonadotropin profile compared to T alone. These data demonstrate that while E_2_ influences gonadotropins in male mice, ablation of aromatase in the brain is not sufficient to disrupt T-dependent regulation of gonadotropins. Taken together, circulating steroid levels in bArKO male mice were consistent with a selective suppression of brain aromatase activity that has systemic consequences on circulating gonadotropins.

## Discussion

Although a large body of high-quality data has been published on the various roles of brain aromatase, the relative contributions to sexual activity of aromatization of T to E_2_ in the testis versus the brain remained unclear because of the absence of a conditional knockout mouse model of the *Cyp19a1* gene to date (19, 20, 27, 30-32). Here, we generated and phenotyped a novel bArKO mouse model with selective disruption of aromatase in the brain and compared hormone levels and sexual activity to a newly generated whole-body tArKO mouse model that showed a severe reproductive phenotype compared to previous reports (26-31). Using the same genetic background of the bArKO and tArKO models, we demonstrated that brain aromatase is, in part, essential in establishing and maintaining appropriate male sexual behavior and steroid hormone balance.

Of critical importance in generating a conditional knockout mouse is the choice of Cre. For generating a brain-specific knockout model, a wide range of neuron-specific Cre-expressing mice are available with varying neuronal recombinase strengths and off-target effects. We chose to use a neuron-specific Cre model that contains the rat nestin promoter (48). In Nestin-Cre mice, Cre expression is detected in neural progenitor cells as early as the embryonic day (E) 10.5-11.5; full recombination throughout the entire central nervous system occurs by E15.5 (48, 49). The time point for Nestin-Cre expression is earlier than that of embryonic peak aromatase expression (E19), resulting in complete elimination of aromatase activity during the neonatal period (50). Nestin-Cre peripheral recombination has only been reported in the heart and kidney, which are nonsteroidogenic tissues, and there are no reports of recombination in the gonads (49, 51). By using Nestin-Cre, we ensured brain-specific aromatase deletion and prevented off-target effects. The hypothalamus is known as an important portion of the brain for sexual activity. Although Nestin-Cre diffusely deletes the aromatase expression in almost all neurons of the nervous system including the hypothalamus, this does not, however, permit us to delineate the specific neuronal types/regions of the hypothalamus that are responsible for a decreased sexual activity in bArKO mice. In the future, we plan to employ mouse Cre models that may allow us to examine specific regions of the hypothalamus or other brain regions that express aromatase.

Using the same *Arom*^*fl/fl*^ mice, we generated whole-body tArKO mice via a cross with the well-characterized Zp3-Cre mice as a positive control. Mouse experiments in general, and behavior experiments in particular, can be affected by genetic background (52-54). To minimize this variable, we created the *Arom*^*fl/fl*^ mice in the C57BL/6J background and all Cre mice were purchased from The Jackson Laboratory on a pure C57BL/6J background. Also, new C57BL/6J breeding pairs were introduced into the colony for at least 10 generations. Previous whole-body ArKO models were created on mixed genetic backgrounds (26-31), and most previously published findings are from mice that were not backcrossed onto a pure background (52-54). The difference in the genetic background of our model might explain sexual behavior and fertility variations observed.

We targeted the untranslated first exon PII and coding exon 2 and partial intron 2 of the aromatase gene. Exon 2 contains the common splice site for all untranslated, tissue-specific first exons as well as the ATG translation start site. It is possible that alternative splicing events upstream of the first exons might result in previously unreported mRNA species. This might lead to truncated protein isoforms of aromatase, which could potentially compromise our model. However, we do not believe that this is likely, given that ATG codons do not exist in any exon 1 of the mouse aromatase gene (11). Additionally, no in-frame ATG codons are observed until exon 5, which if utilized would result in a severely truncated protein. Furthermore, deletion of the DNA sequence between 2 LoxP sites results in a shift of open reading frames. Also, other whole-body ArKO models using our approach to ablate aromatase did not identify any abnormal mRNA or protein isoforms (27).

Our data indicate that brain aromatase is required to regulate T secretion. Both bArKO and tArKO mice had elevated basal serum T but low or absent sexual activity. We posit that the lack of aromatization of T to E_2_ in the brain of bArKO mice diminished the negative feedback of E_2_ on LH secretion, which permitted a rise in T production in the testes. However, we were not able to detect a statistically significant difference in circulating LH and FSH levels between bArKO, tArKO, or control/Het mice. This may be due to the pulsatile nature of LH secretion, which increases the sample-to-sample variation, making it challenging to detect differences between spot-checked levels in circulating blood. Because of the limited accuracy of blood measurements of gonadotropins, this mechanism was not clearly demonstrated. Hormone imbalance was also a characteristic of other previous whole-body ArKO models (31, 55). In addition, our findings are similar to reports of ERα knockout mice or the nonclassical ER knockin mice; for example, ERα knockout males demonstrated a similar approximately 4-fold increase in T despite having unaltered LH levels (3, 56, 57). On the other hand, following castration and hormone replacement, either E_2_ or T was capable of reducing the levels of circulating LH and FSH in both WT and bArKO mice. Thus, these findings collectively suggest that both E_2_ and T regulate gonadotropin secretion in male mice.

As expected, we observed significantly decreased E_2_ in the serum and the brain and testicular tissues of tArKO mice measured by LC-MS/MS. The tArKO mice also lacked sexual behavior and exhibited significantly increased body weight at the age of 20 weeks, indicating E_2_ depletion (26-31, 58). However, there is some residual E_2_ remaining in the serum and tissues of tArKO mice. This phenomenon has also been discerned in the previously published aromatase knockout models (31). In contrast to the previous studies, we employed LC-MS/MS that is much more sensitive and specific for measuring E_2_ compared with antibody-based assays. However, the measurement of the extremely low level of E_2_ is still a challenge in the field (59). There may be a number of potential causes of this phenomenon. It is possible that in addition to the C_18_ steroids, other undefined estrogen-like substances existing in the circulation and tissue of these animals affect the accurate measurement of tissue and serum E_2_ (31). These extremely low E_2_ levels measured by LC-MS/MS in tArKO mice may be influenced by the possible existence of the trace amounts of these estradiol-like substances that may be produced independent of the product of the Cyp19a1 gene (31). It is also possible that T may be converted to E_2_ at very small quantities in tArKO mice via an as yet unknown mechanism or through some low amount of aromatase expression in tArKO mice, which remained below the sensitivity of real-time quantitative PCR.

We demonstrated that brain aromatase contributes to normal sexual behavior in male mice. Intact bArKO mice had an approximately 50% decrease in the number of mounts or intromissions in a 30-minute trial, whereas intact tArKO mice exhibited no sexual activity. Interestingly, the average mount or intromission length (time per event) was not different between intact bArKO mice and controls; however, latency to the initiation of the first sexual activity was prolonged in bArKO mice. This specific phenotype, that is, normal latency, may require localized conversion of T to E_2_ in specific regions of the brain. Overall, conversion of T to E_2_ in the brain plays a role in the initiation and frequency of sexual behavior because estrogen levels are very low in the male circulation. Clearly, T is required for sexual activity because castrated animals demonstrate no sexual activity and T administration restores sexual behavior in castrated WT mice. Further, E_2_ alone is not sufficient to restore normal sexual behavior in castrated mice. In castrated bArKO mice, however, T treatment alone did not result in the restoration of mount or intromission durations; normal sexual activity was restored only upon addition of E_2_ to T treatment. These data indicate that the conversion T to E_2_ in the brain is required for full sexual behavior.

Previous reports demonstrated that whole-body ArKO mice had decreased sexual activity (26-30); the tArKO mice that we generated demonstrated a severe phenotype of complete disruption of sexual behavior. The diminished sexual behavior in ArKO mice has been attributed to a disruption in the organizing role of the aromatization of T to E_2_ during development in the male mice (4, 27, 60, 61). It is proposed that the lack of masculinization leads to a severe disruption in both the initiation and characterization of sexual behavior. While our data do not provide any new insight into the lack of sexual behavior in tArKO mice, it appears that bArKO mice are properly masculinized. They are fertile and exhibit baseline sexual activity greater than that of tArKO males. Furthermore, although our bArKO mice showed diminished sexual activity, they were capable of full sexual activity with hormone replacement therapy. Finally, the qualitative nature of sexual behavior in bArKO mice (time/mount and time/intromission) indicates that bArKO mice have been appropriately masculinized. Programming of brain development to support male sexual behavior in adulthood is known to depend upon perinatal androgen production by the testes and subsequent actions of T and/or its metabolite dihydrotestosterone via androgen receptors, and/or E_2_ aromatized from T, acting through estrogen receptors. Our finding of appreciable masculinization of sexual behavior in bArKO mice suggests that sufficient E_2_ is produced by the neonatal testes to support masculinization of brain circuitries regulating sexual behavior. Alternatively, perinatal T and dihydrotestosterone activation of brain androgen receptors may be sufficient for this process to occur in the mouse.

Relatively few studies have investigated the direct effect of an aromatase inhibitor or estrogen supplementation on male sexual behavior (25, 62, 63). In an elegant study, among groups of men that received T, inhibition of estrogen synthesis by an aromatase inhibitor, as compared with intact estrogen synthesis, was associated with significant decreases in sexual desire and erectile function; these findings provide strong evidence of an independent effect of E_2_ on these measures (63). A separate case study of a castrated man showed that his treatment with estrogen and progestin, possibly in conjunction with minimal androgen levels from his adrenal, is likely responsible for maintaining this patient’s satisfactory sexual drive and functioning (62). In a limited but important study, Carani et al assessed the effects of E_2_ and T administration on a man with aromatase deficiency and hypogonadism (25). Sexual function was limited to masturbation and was seemingly unaffected by T or E_2_ alone; only the combination treatment induced a great increase in libido and in frequency of masturbation and sexual fantasies when both T and E_2_ reached the range of normality (25). This latter report is in agreement with our findings in castrated bArKO mice, whereby full sexual behavior could be restored only by a combination treatment with E_2_ and T (Fig. 3).

In summary, we demonstrated that disrupting aromatase selectively in the mouse brain led to a decrease in sexual behavior, with elevated serum T and decreased brain E_2_. This decreased sexual behavior was restored following systemic administration of E_2_ and T. Our findings support the concept that the mechanism for T-dependent initiation of sexual behavior involves a synergistic activation of the estrogen and androgen signaling pathways in the brain (60). Most importantly, we clearly demonstrate that aromatization of T to E_2_ in the testis and the brain both contribute to normal sexual behavior.

## Abbreviations

A4: androstenedione
ArKO: aromatase knockout
bArKO: brain-specific aromatase knockout
Ct: threshold cycle
CV: coefficient of variation
E#: embryonic day #
E_2_: estradiol
ELISA: enzyme-linked immunosorbent assay
ER: estrogen receptor
ES: embryonic stem (cells)
Het: aromatase heterozygous control mice
FSH: follicle-stimulating hormone
LC-MS/MS: liquid chromatography-tandem mass spectrometry
LH: luteinizing hormone
LLOQ: lower limit of quantitation
PCR: polymerase chain reaction
RIA: radioimmunoassay
RT: reverse transcription
T: testosterone
tArKO: whole-body total aromatase knockout
WT: wild-type

## Additional Information

***Disclosure Summary:*** The authors have nothing to disclose.

***Data Availability:*** All data generated or analyzed during this study are included in this published article or in the data repositories listed in the references.
